# Interactions of *Bacillus subtilis* Basement Spore Coat Layer Proteins

**DOI:** 10.3390/microorganisms9020285

**Published:** 2021-01-30

**Authors:** Daniela Krajčíková, Veronika Bugárová, Imrich Barák

**Affiliations:** Department of Microbial Genetics, Institute of Molecular Biology, Slovak Academy of Sciences, Dúbravská cesta 21, 845 51 Bratislava, Slovakia; daniela.krajcikova@savba.sk (D.K.); veronika.bugarova@savba.sk (V.B.)

**Keywords:** spore coat, protein-protein interaction, cortex, PBP protein, alanine racemase, SpoVM, SpoVD

## Abstract

*Bacillus subtilis* endospores are exceptionally resistant cells encircled by two protective layers: a petidoglycan layer, termed the cortex, and the spore coat, a proteinaceous layer. The formation of both structures depends upon the proper assembly of a basement coat layer, which is composed of two proteins, SpoIVA and SpoVM. The present work examines the interactions of SpoIVA and SpoVM with coat proteins recruited to the spore surface during the early stages of coat assembly. We showed that the alanine racemase YncD associates with two morphogenetic proteins, SpoIVA and CotE. Mutant spores lacking the *yncD* gene were less resistant against wet heat and germinated to a greater extent than wild-type spores in the presence of micromolar concentrations of l-alanine. In seeking a link between the coat and cortex formation, we investigated the interactions between SpoVM and SpoIVA and the proteins essential for cortex synthesis and found that SpoVM interacts with a penicillin-binding protein, SpoVD, and we also demonstrated that SpoVM is crucial for the proper localization of SpoVD. This study shows that direct contacts between coat morphogenetic proteins with a complex of cortex-synthesizing proteins could be one of the tools by which bacteria couple cortex and coat formation.

## 1. Introduction

*Bacillus subtilis* spores develop during a sporulation process that occurs when bacteria find themselves in an environment incompatible with their normal cell cycle. Spores are formed after an asymmetric division of rod-shaped cells from the smaller part of the cell, called the forespore, while the larger mother cell nourishes the spore until it becomes fully mature and can be released into the environment. Spores are known to be exceptionally resistant to a broad variety of conditions. Their resistance arises from the specific arrangement of several protective layers that surround the spore core, which harbors the chromosomal DNA. Two major structures are recognized within the spore: the cortex is a peptidoglycan layer embedded between the inner and outer forespore membranes onto which the spore coat, a thick lamellar protein layer, is formed [1]. More than 80 coat proteins are arrayed in four morphologically distinct coat layers: a basement layer localized directly on the outer forespore membrane, an inner coat, an outer coat and a crust, the outermost layer of the spore [2]. A few coat proteins, the morphogenetic proteins, SpoIVA, SpoVM, SpoVID, SafA, CotE and CotZ, greatly influence coat assembly by controlling the localization of individual proteins on the spore surface and the proper assembly of the four coat layers [3]. SpoIVA and SpoVM form the basement layer of the coat on top of which the inner coat is built under the control of SafA, followed by the outer coat governed by CotE. Assembly of the crust, the coat outermost layer, requires CotZ. The deletion or mutation of any of these proteins produces either coatless spores, or spores whose coat lacks the layer controlled by the missing morphogenetic protein. The basement layer proteins SpoVM and SpoIVA also affect the formation of the cortex [4,5]. These two morphogenetic proteins are synthesized under the control of the σ^E^ transcription factor and are deposited onto the forespore surface very early after the asymmetric septum is formed. SpoVM is a small, 26-amino-acid-long peptide that is inserted into the outer forespore membrane via its amphipathic α-helix, which has an affinity for membranes with a positive curvature [6]. SpoIVA, a structural protein with ATPase activity, directly interacts with SpoVM and undergoes conformational changes after ATP hydrolysis that enable it to polymerize and cover the forespore surface [7,8]. SpoVM and SpoIVA are co-dependent in their localization: through an interaction with SpoVM, SpoIVA reaches the local concentration required for its polymerization on the outer forespore membrane. Initially, SpoVM’s association with the membrane is rather dynamic: it dips into and out of the membrane, but polymerized SpoIVA protein entraps SpoVM into its proper place, to the forespore membrane [9]. SpoIVA recruits SpoVID, a morphogenetic protein which is, together with SpoVM, essential for spore encasement [10]. During this process, individual coat proteins gradually cover the entire forespore surface in multiple waves, all depending on a cascade of transcriptional sigma factors which control the expression of coat components.

SafA, as noted above, directs inner coat formation. SafA has a peptidoglycan-binding LysM domain and the protein is localized in the coat fraction at the interface of the cortex and coat, and also in the cortex layer [11]. The ultrastructure of SafA mutant spores examined by thin sectioning transmission electron microscopy showed that the inner coat was impaired, losing its usual characteristics, and moreover, the cortex, inner coat and outer coat seemed to lack their regular tight adherence [11].

Outer coat formation is directed by CotE, a morphogenetic protein which localizes at the intersection of the outer and inner coat layers. In spores of *cotE* mutant, dark patches of mis-assembled proteins attached to the inner coat were observed [12]. CotE also controls the deposition of the crust layer proteins. Some inner coat proteins, like CotS, were also observed to depend on the presence of CotE [13,14].

Several studies demonstrated that morphogenetic proteins are in direct contact, indicating that they could form a basic, foundational structure, a scaffold to which the coat proteins are attached as the process of spore coat formation proceeds [15,16,17,18,19]. Other coat proteins are also produced at the same time as the key morphogenetic proteins, all of which are under the control of σ^E^. Their role in spore development is often unknown, but the localization some of them have been shown to depend on the key morphogenetic proteins [20]. In the present study, we looked for contacts between the morphogenetic proteins and three coat components which appear to be recruited to the forespore surface during the first waves of coat assembly [20]. One of these is the spore alanine racemase YncD [21]. It is an early expressed protein whose deposition on the forespore surface is CotE-dependent. Alanine racemases catalyze the reversible racemization of l-alanine to d-alanine; they are ubiquitous in bacteria and are crucial for cell wall synthesis since d-alanine is a component of peptidoglycan. The role of YncD during the *B. subtilis* cell life cycle is presently unknown, but the *Bacillus anthracis* spore alanine racemase Alr, which is distributed in the exosporium and coat layer [22,23], protects developing spores against premature germination by converting l-alanine, a germinant, to d-alanine, a germination inhibitor. The function of other two proteins studied here, YhaX and YheD, is unknown.

There are a number of enzymes involved in cortex formation which are also under the control of σ^E^, expressed early in sporulation and are localized to the outer forespore membrane, and they are not coat proteins. A link between coat and cortex formation was discovered by Ramamurthi’s group, which found that a peptide called CmpA is an important factor participating in controlling coat formation [24]. CmpA monitors whether the basement layer consisting of SpoIVA and SpoVM is correctly assembled [25] and ensures that improperly developed spores are destroyed. However, there is no information on what processes take place in the absence of SpoIVA and the molecular mechanism and details of how SpoVM and SpoIVA affect cortex synthesis are still unknown. In this work, we sought to identify interactions between coat morphogenetic proteins and three proteins which are known to be essential for cortex production, SpoVB, SpoVD and SpoVE [26,27,28]. SpoVD, a class B, high-molecular-weight penicillin-binding protein (PBP) with transpeptidase activity, associates with SpoVE, which belongs to the SEDS (shape, elongation, division and sporulation) family of proteins [27]. SpoVE was thought to be a lipid II flippase, but later appeared to function as a glycosyltransferase [29]; the role of flippase is likely to be taken by SpoVB, which is a member of the multidrug/oligosaccharidyl-lipid/polysacharide (MOP) exporter superfamily [30].

In this work, we show that YncD associates with the morphogenetic proteins CotE and SpoIVA. The spores of *yncD* mutant are slightly less resistant to wet heat and can germinate to some level in a trace amount of alanine. We also find that the coat morphogenetic protein SpoVM directly binds SpoVD and that SpoVD localization to the spore surface is SpoVM dependent. Although control of the coat and cortex syntheses is certainly very complex, physical contacts between the key proteins involved could be one of the methods by which cells link the two processes. As a result, SpoIVA and SpoVM have a large impact on coat and cortex formation and spore maturation in general.

## 2. Materials and Methods

### 2.1. Bacterial Strains and Growth Conditions

The genomic DNA of *Bacillus subtilis* strain PY79 [30] was used for the amplification of the *yncD* gene and its upstream and downstream sequences. *Escherichia coli* strain MM294 (*endA1 hsdR17 supE44 thi-1 recA1*) was chosen as the host for the cloning and maintenance of all plasmids. The bacterial two hybrid assay was performed using *E. coli* strain BTH101 (F−, *cya-99, araD139, galE15, galK16, rpsL1 (Str r), hsdR2, mcrA1, mcrB1*) (Euromedex, Souffelweyersheim, France). Recombinant proteins were produced in *E. coli* strain BL21 (DE3) (F−, *ompT, hsdSB, (rB−mB−), gal, dcm*) (Novagen, (Novagen®, Merck KGaA, Darmstadt, Germany). Bacteria were grown in LB medium supplemented with appropriate antibiotics at 37 °C if not otherwise indicated.

### 2.2. Construction of Bacillus Subtilis yncD Strain

To delete the *yncD* gene from the *Bacillus subtilis* chromosome, we prepared the plasmid pUS_deltayncD. The plasmid was constructed by Gibson assembly [31] using four DNA fragments: a 349 bp DNA fragment of the *yncD* upstream sequence, including the first 42 bp of the gene, amplified with primers Gib upstream yncD5′ and Gib upstream yncD3′ from *B. subtilis* genomic DNA; a 1241 bp DNA fragment containing a gene for kanamycin resistance, amplified with primers Gib pUS19kan5′ and Gib pUS19kan3′ from plasmid pUK19; a 345 bp DNA fragment encompassing the downstream sequence of *yncD* and the last 36 bp of the gene, amplified with primers Gib downstream yncD5′ and Gib downstream yncD3′ from *B. subtilis* genomic DNA; and plasmid pUS19 [32], digested with the PstI and EcoRI restriction enzymes. The plasmid pUS_deltayncD was used to transform the *B. subtilis* competent cells. Null mutant clones generated after insertion of the kanamycin cassette by a double cross-over event were tested for spectinomycin sensitivity and verified by PCR; the PCR fragments were also sequenced.

### 2.3. Analysis of Protein–Protein Interactions Using a Bacterial Two Hybrid System (BACTH)

A bacterial two hybrid system based on adenylate cyclase was used [33]. All genes were cloned into four vectors—pUT18, pUT18C, pKT25 and pKNT25—which allowed coat proteins to be fused with the T18 or T25 domains of adenylate cyclase on their N- or C-termini. All genes were amplified by PCR using specific primers Table S1 and *B. subtilis* genomic DNA as the template. After cutting with appropriate restriction enzymes, the genes were cloned into the corresponding sites of the pKNT25, pKT25, pUT18 and pUT18C vectors. All plasmid constructs were verified by restriction analysis and by DNA sequencing. To examine the interactions between the individual proteins, the *E. coli* BTH1 strain was co-transformed with a pair of plasmids encoding the T25 or T18 fusions and transformants were plated onto LB indicator plates, supplemented with antibiotics and containing 40 μg/mL X-gal (5-bromo-4-chloro-3-indolyl-D-galactopyranoside) and 0.5 mM IPTG (isopropyl-β-D-1-thiogalactopyranoside). Bacteria were grown at 30 °C for 48 h. A blue color was observed in those bacterial colonies where direct contact between the expressed hybrid proteins occurred. The interactions were quantified by measuring their β-galactosidase activity using o-nitrophenol-D-galactopyranoside (ONPG) as a substrate. Bacteria grew on LB plates supplemented with antibiotics and 0.5 mM IPTG at 30 °C for 45 h. Bacterial colonies were scraped off, lysed with chloroform and SDS and the β-galactosidase activity was determined as described by Miller [34].

### 2.4. Protein Expression and Purification and Pull-Down Assay

To express His-tagged recombinant YncD, we used an expression plasmid based on pET28a or pETDuet vectors (Novagen). The *yncD* gene was amplified with specific primers Table S1 from *B. subtilis* genomic DNA and, after cleavage with restriction enzymes, cloned into the corresponding cloning site of a plasmid. To express un-tagged CotE and SpoIVA, the genes were amplified by PCR with the appropriate primers Table S1 using *B. subtilis* genomic DNA as a template, digested with the appropriate restriction enzymes and cloned into a pETDuet plasmid cut with corresponding restrictases; the proteins were then produced as before [35]. Briefly, *E. coli* BL21 (DE3) cells transformed with expression plasmids were grown in LB medium supplemented with antibiotics at 37 °C until the culture reached an OD600 of ≈0.6 and then induced by the addition of 1 mM IPTG. The cells were collected after 3 h of additional cultivation and stored at −80 °C until used. The YncD protein was isolated in a 50 mM Tris/HCl buffer, pH 8, containing 150 mM NaCl. Cells were lysed by sonication and the lysate was centrifuged at 70,000× g for 30 min and the protein was purified by metal affinity chromatography using a His Trap^TM^ high performance (HP) column ( Merck KGaA, Darmstadt, Germany). For the pull-down assay, cells expressing the proteins were sonicated and the bacterial lysates were mixed and incubated on ice for approx. 1 h. After centrifugation, the proteins were co-purified using a Ni-agarose column. Interacting proteins were separated by sodium dodecyl sulfate-polyacrylamide gel electrophoresis (SDS-PAGE) and identified by Western blotting using polyclonal antibodies [35].

### 2.5. Spore Preparation

Spore crops were prepared by sporulation on Difco sporulation media (DSM) plates [36]. A single colony was used to inoculate LB medium and cultivated overnight at 37 °C. This culture was then used to inoculate liquid DSM and the bacteria were grown until OD600 = 0.8. 200 μL of this culture was spread on DSM plates and left at 37 °C for 3 days, followed by growth at room temperature for another 4–5 days. Spores were harvested, purified by the intensive washing with ice-cold water to remove cell debris and vegetative cells and stored at 4 °C until use.

### 2.6. Germination Assay

Before germination, spores were heat activated at 70 °C for 30 min followed by cooling on ice. Spores were resuspended in PBS buffer to reach an optical density OD600 = 1.0 and L-alanine was added to final concentrations of 100 mM, 1 mM or 0.01 mM. Germination was monitored by the loss of optical density at 600 nm at room temperature for up to 120 min.

### 2.7. Thermal Resistance Assay

The thermal resistance assay was performed in PCR Eppendorf tubes using a dry bath system. Spores (approx. 1 × 10^8^ CFU/ mL) were suspended in PBS buffer and heated at 85 °C for 15 min to inactivate all vegetative or germinating spores. The wet heat resistance of at least three independent spore preparations was measured at 100 °C and 110 °C. 20 μL aliquots of spore suspension were used and at least 5 time points were performed for each spore prep and temperature. The Eppendorf tubes were immediately cooled on ice, the tube contents were transferred into 480 μL PBS and serial 10-fold dilutions were made to monitor the number of surviving spores by counting the bacterial colonies growing on LB plates. The *D*-value (the decimal reduction time or time required to kill 90% of the spores) was determined from a linear regression of survivors (log10 CFU/mL) versus exposure time.

### 2.8. Fluorescence Microscopy

The localization of mCherry-SpoVD in sporulating wild-type and *spoVM* mutant cells was visualized by fluorescence microscopy. Cells grown in DSM were examined three or five hours after reaching stationary phase. The samples were spotted onto a poly-L-lysine glass slide and covered with a poly-L-lysine coverslip. All images were obtained using an Olympus BX63 microscope equipped with an Andor Zyla 5.5 sCMOS camera (Olympus Europa SE & Co. KG., Hamburg, Germany). Olympus CellP imaging software was used for image acquisition and analysis. The final adjustment of the fluorescent images was done using Fiji ImageJ (open source software).

## 3. Results

### 3.1. Examination of Protein-Protein Interactions Using a Bacterial Two Hybrid System

To find potential direct contacts between different sets of proteins, we employed a bacterial two hybrid system. Previously, we performed a systematic study of potential interactions between the outer coat and crust proteins [31] and discovered a very complex protein interaction network within these coat layers. Here we investigated the interactions of morphogenetic coat proteins with three proteins, YncD, YhaX and YheD. We also examined three proteins essential for spore cortex synthesis: SpoVD, SpoVE and SpoVB.

**Figure 1:**
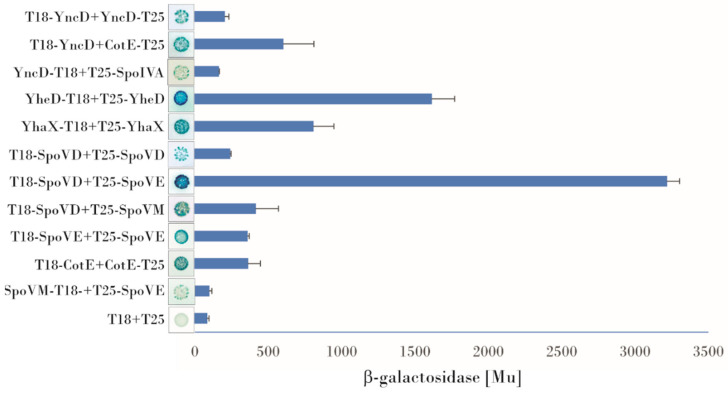
Bacterial two hybrid analysis of the interactions of spore proteins. The combinations of plasmids that provided the most intense signals on X-gal plates are shown. The β-galactosidase activity was determined as the average of at least three independent cell samples. All β-galactosidase values were subjected to the Dixon outlier test (*p* < 0.01).

Protein Interactions of YncD, YhaX and YheD:

- YncD contacts with morphogenetic coat proteins: YncD, an alanine racemase, is deposited onto a developing forespore under the control of CotE, as reported by McKenney [2]. Using BACTH, we observed that YncD physically contacts CotE. Screening all possible combinations of plasmids with the *cotE* and *yncD* genes fused to either the T18 or T25 domains of adenylate cyclase in plate assays showed that the highest expression of the β-galactosidase reporter gene arose from the T18-YncD/CotE-T25 combination (600 Mu). Some signals were also obtained for the combinations T18-YncD/T25-CotE, T25-YncD/CotE-T18, and T25-YncD/T18-CotE. As described previously [31], we compared these signals with the T18-CotE/CotE-T25 self-interaction used as the positive control (360 Mu in these experiments) [18,32]. The strength of the interaction of YncD and CotE observed by BACTH was almost twice that of CotE self-interaction and the observed blue color of the bacterial colonies harboring the YncD/CotE pair after 48 h of incubation was also more intense. A direct interaction between SpoVID or SpoVM and YncD was not detected, but a weak blue color could be observed in those bacterial colonies harboring combinations of plasmids containing the *spoIVA* and *yncD* genes. The highest β-galactosidase signal detected on X-gal plates for this pair was obtained from the YncD-T18/T25-SpoIVA and T18-SpoIVA/YncD-T25 combinations (160 Mu), which was approximately twice the negative control. A YncD/YncD self-interaction was also observed (220 Mu), indicating that the protein can form oligomers (Figure 1).
- Protein contacts of YheD and YhaX: Although the deposition of these two envelope proteins on the forespore surface has been shown to be dependent on SpoIVA [20], our bacterial two hybrid assays did not detect any direct binding to SpoIVA or to any other morphogenetic protein, only an YheD self-interaction could be observed. The positive signal was one of the strongest in this screening (1724 Mu) and arose from two plasmid combinations (T25–YheD/YheD–T18 and T25–YheD/T18–YheD). YhaX also formed homooligomers: β-galactosidase reporter gene activity was measured after complementation of the hybrid proteins T25–YhaX/YhaX–T18 and T25-YhaX/T18–YhaX (704 Mu) (Figure 1).
- Bacterial two hybrid assay of interactions of SpoVB, SpoVD and SpoVE with coat morphogenetic proteins: BACTH was previously used to successfully investigate interactions between protein involved in cell wall synthesis [33], many of which are integral membrane proteins or are associated with a membrane. Here we investigated the possible contacts between proteins essential for cortex peptidoglycan synthesis. An analysis of the topology of these proteins predicted that SpoVE and SpoVB have 10–15 transmembrane segments while SpoVD has one transmembrane segment at its N-terminus. Despite some difficulties with cloning, especially of SpoVE and SpoVD which may be toxic to *E. coli* cells, we managed to prepare the correct plasmid constructs. First, we tested their self-association. No homodimerization of SpoVB was observed, but positive signals were obtained for SpoVE and SpoVD; in both cases, β-galactosidase expression occurred in only one combination of plasmids. For SpoVE, we saw that bacterial colonies turned blue when the SpoVE–T18/T25–SpoVE hybrid proteins were co-expressed and we measured a β-galactosidase activity of up to 360 Mu (Figure 1). For SpoVD, it was important that, after fusion with the adenylate cyclase fragment, the protein be properly incorporated into the membrane such that the T18/T25 domain would be in the cytoplasm (on its N-terminus). Under these restrictions, we detected a positive signal from the combination T18–SpoVD/T25–SpoVD whose β-galactosidase activity reached 240 Mu (Figure 1). We next examined the interactions of these proteins with the coat morphogenetic proteins. Our experiments showed that SpoVD interacts with SpoVM when the hybrid protein T25–SpoVD was combined with T18–SpoVM; very faint blue bacterial colonies were also observed when T25–SpoVD was co-expressed with SpoVM–T18. Quantitative evaluation of the contact strength showed that the interaction of SpoVD with SpoVM was at roughly the same level as the CotE self-interaction (420 Mu vs. 360 Mu), which makes it an intermediate-strength interaction. Screening the interactions of SpoVE with the coat morphogenetic proteins indicated that there may also be an interaction of SpoVE and SpoVM, although the positive signals were very weak and could be identified only after prolonged incubation of the X-gal plate at 4 °C. The signals could be detected only for the T25–SpoVE and SpoVM–T18 and T18–SpoVM combinations (the β-galactosidase activity was approximately twice the negative control). The BACTH system did not detect any contact between these proteins and SpoIVA or between SpoVB and SpoVM or SpoIVA (Figure 1).

### 3.2. Examination of the YncD–CotE and YncD–SpoIVA Interactions Using a Pull-Down Assay

**Figure 2:**
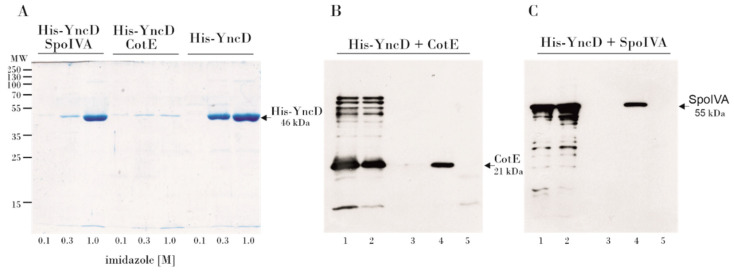
Pull-down analysis of the YncD interaction with morphogenetic proteins. (**A**) Sodium dodecyl sulfate-polyacrylamide gel electrophoresis (SDS-PAGE) gel of proteins co-expressed in *Escherichia coli*, loaded onto a HisTrap HP Ni-column and eluted with an imidazole step gradient. The imidazole concentration is shown in the figure. Lanes 1–3 contain fractions eluted after co-expression and co-purification of YncD+SpoIVA; lanes 4–6 contain fractions eluted after co-expression and co-purification of YncD+CotE; lanes 7–9 show fractions eluted after expression and purification of YncD alone. In all experiments we used the same volume of the same cell extract. (**B**) Western blot analysis of the YncD–CotE interaction. Proteins were probed with a polyclonal anti-CotE antibody. Lane 1–cell lysate with over-expressed, untagged CotE; lane 2–a mix of cell lysates with over-expressed CotE and His-tagged YncD; lane 3–negative control, elution from Ni-column, untagged CotE did not bind to the column; lane 4–CotE co-purified with His-tagged YncD; lane 5–His-tagged YncD does not provide a signal when using the anti-CotE antibody. (**C**) Western blot analysis of the YncD–SpoIVA interaction. Cell lysates with over-expressed proteins were mixed, loaded onto a HisTrap HP Ni-column and eluted with imidazole. Proteins were probed with a polyclonal anti-SpoIVA antibody. Lane 1–cell lysate with over-expressed SpoIVA; lane 2–mix of cell lysates with over-expressed SpoIVA and His-tagged YncD; lane 3–negative control, elution from Ni-column, untagged SpoIVA did not bind to the column; lane 4–SpoIVA co-purified with His-tagged YncD; lane 5–His-tagged YncD does not give a signal from an anti-SpoIVA antibody.

To verify the physical contacts between YncD and CotE and SpoIVA, we performed a pull-down assay. We co-expressed His-tagged YncD and untagged CotE in a pETDuet plasmid. Co-purification of the tagged YncD and untagged CotE proteins was performed using nickel-affinity chromatography. The solubility of recombinant YncD when co-expressed together with CotE was unexpectedly greatly reduced, and the yield of soluble proteins after chromatography on the Ni-column was relatively low (Figure 2A). It seems likely that the complex formed by the two proteins during co-expression was less soluble than the proteins themselves. To circumvent this problem, both proteins were expressed separately, the bacterial lysates were mixed, applied to a Ni-column and the proteins were eluted with imidazole. In the elution fractions, a faint band of pulled-down CotE was visible on an SDS-PAGE gel after Coomasie blue staining (not shown). In addition, we identified the untagged CotE by Western blotting using a polyclonal anti-CotE antibody (Figure 2B). It is clear that untagged CotE was eluted from the column only in the presence of His-tagged YncD, whereas in the absence of YncD no CotE protein was detected in the elution fractions, thus confirming the YncD–CotE interaction observed in the bacterial two hybrid experiment.

Similarly, we performed a pull-down assay of the YncD–SpoIVA interaction. Lysates containing untagged SpoIVA were mixed with His-tagged YncD and the proteins were purified on a Ni-column. We showed by Western blotting using a polyclonal anti-SpoIVA antibody that SpoIVA was eluted from the column in the presence of His-tagged YncD. Untagged SpoIVA did not bind to the Ni-resin on its own. The formation of a complex between SpoIVA and YncD is also indicated by a significant drop in the solubility of YncD in the presence of SpoIVA, although not as dramatic as in the case of CotE (Figure 2C).

### 3.3. Characterization of the ΔyncD Mutant Spores

BACTH experiments and pull-down assays showed that YncD can physically associate with two morphogenetic proteins, so we investigated whether deletion of its gene would cause any changes in spore properties. When growing in sporulation media, we did not see any significant difference between the *ΔyncD* and wild-type strain and the sporulation frequencies were essentially the same. Their resistance to chloroform was also identical (data not shown). In addition, when spores were treated with lysozyme and hydrogen peroxide, the percentage of survivals did not differ significantly Table 1.

**Figure 3:**
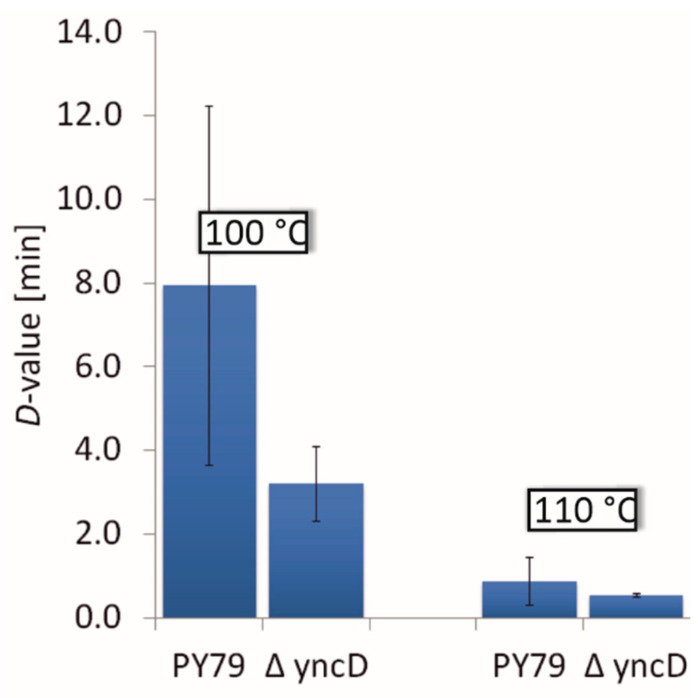
Heat resistance of wild-type and *ΔyncD* spores. The average D-values calculated for 100 °C and 110 °C are shown.

Additionally, the wet heat resistance of the purified spores was examined and the decimal reduction time or *D*-value (the time for 90% of the population to die) of the wild-type and mutant strains were determined. It was found that the heat resistance of the *ΔyncD* mutant was slightly lower than that of the PY79 strain measured at 100 °C (average *D*-value of three different *yncD* spore preparations, 3.2 min; wild-type spores, 8.0 min), at 110 °C the *D*-values for both strains practically did not differ (Figure 3, Table 2).

**Figure 4:**
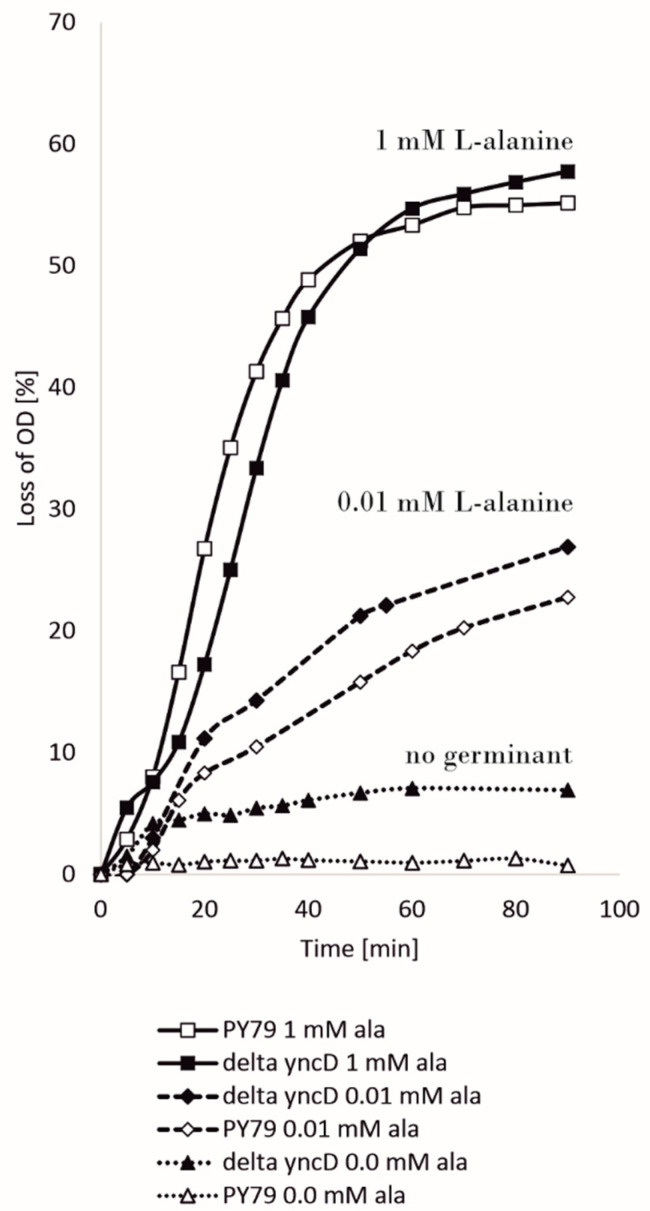
Germination assay. Spores were heat activated as described in Material and Methods, induced to germinate by l-alanine in PBS buffer and the OD_600_ was monitored. The assays were repeated at least four times using independent spore preparations.

Another characteristic that may suggest a role for YncD in bacteria is germination. After heat activation at 70 °C, spores were induced by l-alanine in concentrations of 100 mM, 1 mM and 0.01 mM, and germination was monitored by the decrease in OD_600_. Figure 4 shows a typical progression. The spores germinated similarly at the highest concentration of alanine; at 1 mM and 100 mM alanine concentration, only a subtle difference between the lag phases of the wild-type and mutant spores could be observed; at micromolar alanine concentrations, *ΔyncD* spores germinated faster than wild-type spores. We also noticed that mutant spores germinated slightly even at zero l-alanine concentration, while no germination at all could be observed in the wild-type strain. We also looked for a phenotype observed in studies of the spore-associated alanine racemase of *B. anthracis* where it was shown that spores began to germinate prematurely in the mother cytoplasm in the absence of the enzyme [23] (Chesnokova et al., 2009), although no similar effect was seen under the conditions we used.

### 3.4. Localization of SpoVD-mCherry is Dependent on SpoVM

**Figure 5:**
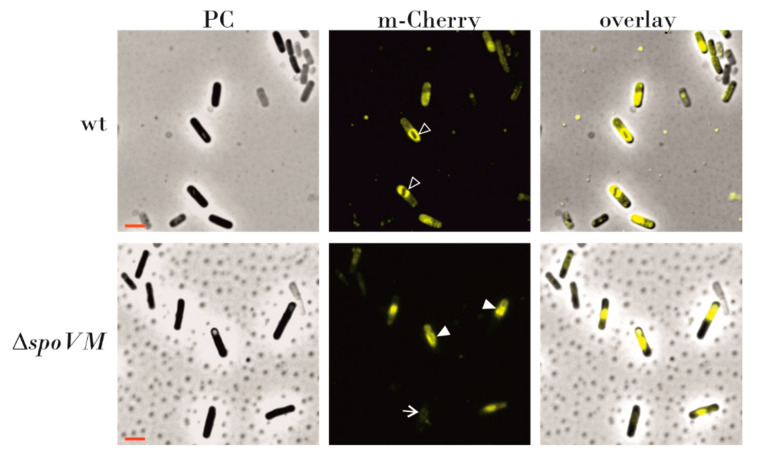
Localization of mCherry-SpoVD in sporulating cells. Cells grown in Difco sporulation media (DSM) were examined four hours after reaching stationary phase. The left panels show phase contrast images (PC); the middle panels show m-Cherry detection (false colored yellow); the right panels are merged images of PC and m-Cherry. The mCherry-SpoVD signal is around the forespore (open arrow heads) in wild type cells. In *spoVM* mutant cells, the fusion protein signals are mis-localized in the mother cell as clumps of proteins which are sometimes attached to the forespore (white arrowheads). The complete white arrow shows the proper localization of the mCherry-SpoVD protein. The red scale bar represents 2 μm.

Fay et al. [27] proposed that SpoVD is recruited to the spore surface by SpoVE, which is subsequently stabilized by SpoVD. To investigate whether the interaction of SpoVD with SpoVM also affects the localization of SpoVD on the spore surface, we used *B. subtilis* strain IB1776, with a C-terminal fusion of SpoVD with mCherry [34] inserted into the *amyE* locus under the native *spoVD* promoter, and strain IB1790 with *spoVD-mCherry* and a disrupted *spoVM* gene. We found that after four hours of sporulation, in strain IB1776 SpoVD-mCherry was enriched in the forespore membrane, and later completely surrounded spore surface (Figure 5), whereas in strain IB1790 the fluorescence signal was observed mostly in the mother cell cytoplasm and localization in the forespore membrane occurred in only 20–30 % of the 216 cells observed. In addition, in about 30 % of cells SpoVD-mCherry was seen as a clump of protein close to the forespore (Figure 5).

## 4. Discussion

**Figure 6:**
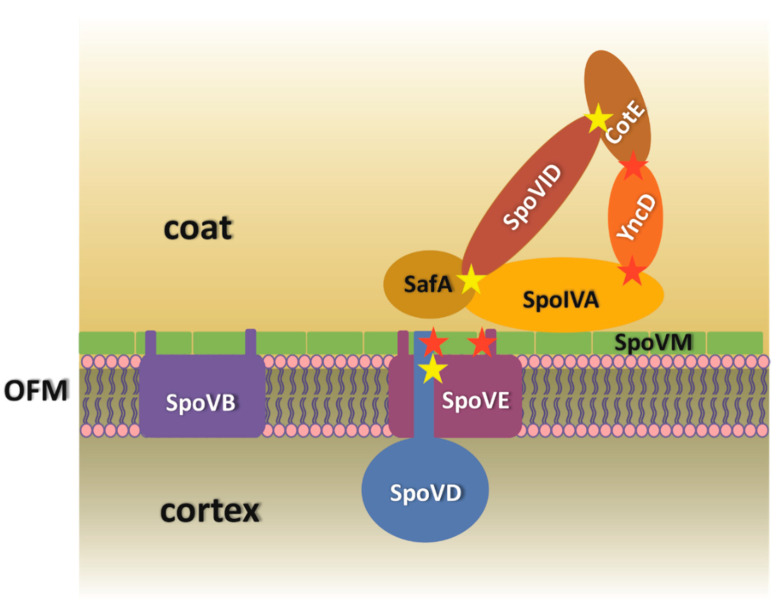
Model of the interactions between the coat proteins and the cortex synthesizing proteins. SpoIVA together with SpoVM, SpoVID, SafA and CotE build the basic structure of the spore coat through direct interactions. YncD is in contact with two of the morphogenetic proteins, SpoIVA and CotE. The localization of the SpoVD–SpoVE protein complex is dependent on SpoVM. The N-terminal domain (SpoVD) or N- and C-terminal domains (SpoVE) form physical contacts with SpoVM. The interactions discovered in this work are marked with red stars, previously known interactions are marked with yellow stars. The Bacterial Two Hybrid System (BACTH) assay did not reveal any physical contacts between SpoVB and these proteins. (OFM—outer forespore membrane.)

In this work we have investigated contacts between the basement layer morphogenetic proteins SpoVM and SpoIVA, the coat proteins which are synthesized at the onset of coat formation and some proteins essential for cortex synthesis. The initial stages of coat assembly require both these proteins and, as mentioned above, they interact with each other to form a platform for the deposition of the coat layers. The molecular mechanism of the contact between these two proteins has been described in detail and the last model of Peluso et al. [9] explains the process of their localization on the spore surface as the coordination of several events. According to this model, SpoVM localizes on the outer forespore membrane, followed by the recruitment of SpoIVA through its contact with SpoVM and the subsequent ATP-dependent polymerization of SpoIVA. The highly cross-linked SpoIVA then stitches SpoVM onto the membrane. In the present work, we asked if other coat proteins which are expressed early during spore coat formation and are not among the morphogenetic proteins, also interact with SpoIVA or SpoVM. Specifically, we were interested in three proteins, YhaX, YncD and YheD. YhaX is a protein of unknown function whose expression is induced from a σ^E^ controlled promoter in response to phosphate starvation [35]. It was shown that this protein localizes to the nascent spore coat under the control of SpoIVA in the first wave of coat encasement. Using a BACTH system we did not detect any direct contacts to morphogenetic proteins, but YhaX was self-interacting, suggesting that it might form larger oligomers on the spore surface. Similarly, the function of YheD in *B. subtilis* cells has not yet been determined, although localization studies have clearly shown that it forms a shell around the spore [36]. The way in which YheD surrounds the spore surface is unusual: the protein initially forms two discrete circles with the one on the mother side of the forespore being more robust, and it later covers the entire surface of the spore. We observed a strong self-interaction for YheD and it is likely that the distribution of this protein throughout the spore surface occurs via polymerization of the protein itself. No interactions between YheD and the other tested proteins were observed. It may be that the association of YheD with proteins within the spore coat requires specific steric conditions which cannot be produced in the heterologous host used for the BACTH system. We showed that the third examined protein, the spore alanine racemase YncD, interacted with two morphogenetic proteins, CotE and SpoIVA (Figure 6). Although the localization of YncD to the spore surface was found to depend on CotE by McKenney et al. [20], their mutual interaction has not been previously reported. The association of CotE with YncD appears to be one of the strongest CotE interactions that we have discovered, including those in our earlier study of CotE contacts [31]. We have also found that the complex which these proteins form is almost insoluble, although both proteins are well soluble individually. This accords with our earlier study [16], in which we observed a tendency for coat proteins to change their solubility after co-expression in *E. coli*, indicating the possible formation of large polymeric structures.

More questions are raised by our discovery of the YncD–SpoIVA interaction. It is well known that SpoIVA directly or indirectly governs the localization of all coat proteins on the spore surface. However, except for the morphogenetic proteins SpoVM, SpoVID and SafA, no other physical contacts of SpoIVA have been found to date. Could YncD, part of the outer coat layer [20], be in contact with SpoIVA, which is a basement layer protein? Is it possible that the YncD interaction with SpoIVA is transient and exists just at the beginning of coat formation or could YncD be a component of more than one coat layer? Lastly, the exact role of YncD is not known. YncD seems to have no crucial structural function, since deletion of its gene does not significantly impair the coat’s protective properties and such spores were still resistant to a hydrolyzing enzyme and oxidative stress. To understand why bacteria have a spore-specific alanine racemase, we also studied the germination process of mutant and wild-type spores. Butzin et al. [37] observed that with increasing alanine concentration, the germination of the strain in which both alanine racemase genes were deleted, the vegetative *dal* (*alrA*) and the spore protein *yncD* (or *alrB*), did not differ compared to wild-type spores. However, it is puzzling that in our experiments, spores of *yncD* mutant germinated at a slow rate even without the addition of alanine, whereas no germination was observed for wild-type spores. We hypothesize that germination of the mutant strain under this condition occurred as a result of the presence of a trace remnant of alanine. While YncD racemase in wild-type spores inhibits germination by converting l-alanine to D-alanine, residual alanine could initiate some germination of the mutant spores. Perhaps YncD is important in the non-laboratory, natural environment for monitoring whether the conditions for germination are optimal. At very low concentrations of alanine, YncD blocks this process. This function perhaps cannot be observed at the higher alanine concentrations normally used in germination assays.

Given the contact between YncD and SpoIVA, we also considered whether the production of d-alanine, a peptidoglycan component, by YncD might be important for cortex synthesis. The cortex maintains spore dormancy by keeping the spore core dehydrated and thus resistant to heat and other physically damaging factors. We therefore tested the thermal resistance of *yncD* spores. We did not detect significant differences between the heat resistance of wild-type and *yncD* spores at lower temperatures, but *yncD* spores were less resistant against wet heat at 100 °C. Nevertheless, the deletion of the *yncD* gene did not have a dramatic effect on the heat resistance of the spores and we conclude that the activity of the vegetative *alrA* (dal) alanine racemase produces sufficient d-alanine for cortex peptidoglycan. To date, only a few genes have been identified that alter the cortex formation to such an extent that heat resistance changes radically. These include *spoVD*, *spoVE* and *spoVB*, whose deletion entirely stops or considerably diminishes peptidoglycan production. Here, we asked whether SpoIVA-SpoVM controls cortex synthesis simply by attaching these cortex-synthesizing proteins to the spore surface? It has long been known that the absence of SpoIVA or SpoVM impairs the assembly of both protective structures [4,5]. The mechanism behind this is not clear, although as mentioned above, *B. subtilis* blocks cortex formation using the small peptide CmpA if the spore coat is misassembled [38]. Therefore, we examined whether SpoVD, SpoVE and SpoVB interact with SpoIVA and SpoVM. We found that SpoVD physically contacts SpoVM (Figure 6) with a moderately strong interaction. After finding that they associate in *E. coli*, we tested whether they were in contact on the spore surface and we looked whether the localization of SpoVD-mCherry in wild-type and mutant cells differed. We found that the deposition of SpoVD-mCherry on the outer forespore membrane is dependent on SpoVM, confirming the observation obtained by BACTH analysis. The fluorescence signal in *spoVM* mutant cells was often mislocalized and formed a large clump in the mother cell cytoplasm that stuck to the forespore. In contrast, in wild-type cells SpoVD-mCherry nicely encircled the forespore, especially when spores entered the bright phase of spore development. These results indicate that the protein–protein interactions between SpoVM and SpoVD are important for anchoring SpoVD to the outer forespore membrane. Hence, it is reasonable to assume that the proper placement of the basement spore coat layer is necessary for the localization of key cortex synthesizing proteins. The mechanism by which cells control cortex and coat formation is certainly more sophisticated and interconnected than originally thought. Recent works by Fernandes et al. [25] and Pereira et al. [11] showed that SafA, an inner coat morphogenetic protein, is also recruited to the cortex layer and it affects the adherence of the cortex and coat layers. Moreover, the localization of SafA at the spore surface is influenced by SpoVE. However, the mechanism by which SafA is transferred through the outer forespore membrane and what the role of SafA is within the cortex remain to be determined.

## 5. Conclusions

In conclusion, our studies show that the spore alanine racemase YncD is associated with two morphogenetic proteins, CotE and SpoIVA. We propose that YncD affects spore germination at low concentrations of alanine. We also demonstrated a direct contact between SpoVM and SpoVD and suggested that the SpoVM/SpoIVA coat morphogenetic proteins could contribute to the proper localization of the proteins necessary for cortex synthesis, thereby linking the processes of cortex and coat formation. Unquestionably, further studies are needed to elucidate the details of the effect of coat morphogenetic proteins on cortex synthesis.

## Figures and Tables

**Table 1:** Resistance of wild-type and *ΔyncD* spores under various conditions. For resistance assays, spores were treated with lysozyme (1 mg/mL) in PBS buffer for 30 min at 37 °C and 15% H_2_O_2_ in PBS for 10 min. Resistance of spores was followed as the number of colonies forming units before and after treatment.

Strain	Lysozyme			H_2_O_2_		
	Spore count (CFU/mL)		Survival (%)	Spore Count (CFU/mL)		Survival (%)
	−	+		−	+	
PY79	1.1 × 10^8^	2.6 × 10^7^	24	4.2 × 10^7^	1.5 × 10^7^	36
*ΔyncD*	6.6 × 10^7^	1.3 × 10^7^	20	7.8 × 10^7^	1.3 × 10^7^	17

**Table 2:** Heat resistance of the wild-type and *ΔyncD* spores. *D*-values were calculated using a log-linear model for three independent spore preparations at 100 °C and 110 °C. R^2^ is a regression statistic which reflects how well the regression predictions agree with the real data. The closer this value is to the value 1, the closer the measured points are to the calculated ones.

Strain	Temperature	Prep 1		Prep 2		Prep 3	
		D-Value (min)	R^2^	D-Value (min)	R^2^	D-Value (min)	R^2^
PY79	100 °C	5.4	0.96	5.6	0.90	12.9	0.87
	110 °C	1.2	0.87	0.4	0.81	0.4	0.78
*ΔyncD*	100 °C	2.9	0.94	2.5	0.90	4.2	0.94
	110 °C	0.6	0.97	0.6	0.93	0.5	0.80

## Supplementary Materials

The following are available online at https://www.mdpi.com/2076-2607/9/2/285/s1, Table S1: Oligonucleotides, Table S2: Plasmids and strains.
