# Supplementary material for: Interactions of Bacillus subtilis Basement Spore Coat Layer Proteins
Source: Microorganisms. 2021 Jan 30;9(2):285. doi: 10.3390/microorganisms9020285 (PMC7911427; doi:10.3390/microorganisms9020285)
Supplement: Supplementary file 1 [file microorganisms-09-00285-s001.pdf]

microorganisms

MDPI

Supplementary Materials: Interactions of *Bacillus subtilis* Basement Spore Coat Layer Proteins

Daniela Krajčíková, Veronika Bugárová and Imrich Barák *

Table S1. Oligonucleotides.

Primer	Header	Sequence 5'-3'
spoVMpKTN5'	forward primer for cloning *spoVM* into pKTN25, pKT25, pUT18, pUT18C plasmids	GAGAGAGAGGATCCCAAATTTTA CACCATTAAATTGCCGAAG
spoVMpKTN3'	reverse primer for cloning *spoVM* into pKTN25, pUT18, pUT18C plasmids	GAGAGAGAGAATTCCTATCTTTTC TAAATGAGCCCAGC
spoVMpKT3'	reverse primer for cloning *spoVM* into pKT25 plasmid	GAGAGAGAGAATTCATCTTTTCTA AATGAGCCCAGC
spoIVApKNT5'	forward primer for cloning *spoIVA* into pKTN25, pUT18, pUT18C plasmids	GTGTGTGTCTGCAGCGAAAAGGTC GATATTTTCAAGG
spoIVApKNT3'	reverse primer for cloning *spoIVA* into pKTN25, pKT25, pUT18, pUT18C plasmids	GTGTGTGTGGATCCGACAGGATGA TGGCGATTAAG
spoIVApKT5'	forward primer for cloning *spoIVA* into pKT25 plasmid	GTGTGTGTCTGCAGCGGAAAAGGT CGATATTTTCAAGG
pKNTyncD5'	forward primer for cloning *yncD* into pKTN25, pKT25, pUT18, pUT18C plasmids	CGTAGCGCGGATCCGATAAAGCTT TGCCGAGAAG
pKNTyncD3'	reverse primer for cloning *yncD* into pKTN25, pUT18, pUT18C plasmids	CGTAGCGCGAATTCAACACGTATA ATACTGGAGTTG
pKTyncD3'	reverse primer for cloning *yncD* into pKT25 plasmid	CGTAGCGCGAATTCCACGTATAATACTGG AGTTGATAC
pKT25Yhax5´	forward primer for cloning *yhaX* into pKT25 plasmid	GGCGGCTCTGCAGCCTCAAAACA ATTGCTTGCCTTAAAT
pKT25Yhax3´	reverse primer for cloning *yhaX* into pKTN25, pKT25, pUT18, pUT18C plasmids	GGCGGCTGGATCCGGCACACGTTT CATGTGGAACTT
pKNT25Yhax5´	forward primer for cloning *yhaX* into pKTN25, pUT18, pUT18C plasmids	GGCGGCTCTGCAGCTCAAAACAAT TGCTTGCCTTAAAT
pKT25yheD5´	forward primer for cloning *yheD* into pKTN25, pKT25, pUT18, pUT18C plasmids	GGCGGCTGGATCCCAATCCTAAAC GGTTTTTGATCG
pKT25yheD3´	reverse primer for cloning *yheD* into pKTN25, pKT25, pUT18, pUT18C plasmids	GGCGGCTGGTACCGAAGGCCACA ATGCTTCC
pKNTspoVD5'	forward primer for cloning *spoVD* into pKTN25, pKT25, pUT18, pUT18C plasmids	GAGAGAGAGGATCCCCGCGTCTC GAATGTAACG
pKNTspoVD3'	reverse primer for cloning *spoVD* into pKTN25, pUT18, pUT18C plasmids	GAGAGAGTGAATTCTTATCGGCTG CCTCCTTTTC

pKTspoVD3'	reverse primer for cloning *spoVD* into pKT25 plasmid	GAGAGAGTGAATTCTTAATCGGCTGCCTCCTTTTC
pKNTspoVE5'	forward primer for cloning *spoVE* into pKTN25, pKT25, pUT18, pUT18C plasmids	GAGAGTGTGGATCCCACTACTAAAAAAACATCGCCTGATTTG
pKNTspoVE3'	reverse primer for cloning *spoVE* into pKTN25, pUT18, pUT18C plasmids	GAGAGAGTGAATTCCAATACCTCGAGTAGCGGCTTAC
pKTspoVE3'	reverse primer for cloning *spoVE* into pKT25 plasmid	GAGAGAGTGAATTCTCAATACCTCGAGTAGCGGCTTAC
pKNTspoVB5'	forward primer for cloning *spoVB* into pKTN25, pKT25, pUT18, pUT18C plasmids	GAGAGAGAGGATCCAGCGAAACAGACGTTTTTAAAGG
pKNTspoVB3'	reverse primer for cloning *spoVB* into pKTN25, pUT18, pUT18C plasmids	GAGAGAGAGAATTCAGTCGGATGATCAACCGTCC
pKTspoVB3'	reverse primer for cloning *spoVB* into pKT25 plasmid	GAGAGAGAGAATTCTCGGATGATCAACCGTCC
Gib upstream yncD5'	forward primer for amplification of *yncD* upstream sequence for Gibson assembly	ACGCCAAGCTTGCATGCCTGCAGTAACAACGGTAAGTATTTCCTG
Gib upstream yncD3'	reverse primer for amplification of *yncD* upstream sequence for Gibson assembly	TTAGTACCTAGATTTAGATGTCTAACTGCAGAAGATTGACTTCGATCCAAAC
Gib pUS19kanrev5'	forward primer for amplification of *kan* gene for Gibson assembly	AGTTTGGATCGAAGTCAATCTTCTGCAGTTAGACATCTAAATCTAGGTAC
Gib pUS19kanrev3'	reverse primer for amplification of *kan* gene for Gibson assembly	ACTGGAGTTGATACTTTAAAAATCGGATCCACCAAGACGAAGAGGATGAAG
Gib downstream yncD5'	forward primer for amplification of *yncD* downstream sequence for Gibson assembly	CTTCATCCTCTTCGTCTTGGTGGATCCGATTTTTAAAGTATCAACTCCAG
Gib downstream yncD3'	reverse primer for amplification of *yncD* downstream sequence for Gibson assembly	TGTAAAACGACGGCCAGTGAATTCGTGATCGGTTTTGCTTTCC
yncDpETDuet5'	forward primer for cloning of *yncD* into pETDuet	GAGAGAGGGATCCGATAAAGCTTTGCCGAGAAGTTTG
yncDpETDuet3'	reverse primer for cloning of *yncD* into pETDuet	GAGAGTCGAATTCCTACACGTATAATACTGGAGTTG
spoIVA5′	forward primer for cloning of N-terminal region of *spoIVA* into pETDuet	AGGAGGAGGCATATGGAAAAGGTCGATATTTTCAAGG
spoIVA3′	reverse primer for cloning of C-terminal region of *spoIVA* into pETDuet	GCTGCTCTCCTCGAGTTACAGGATGATGGCGATTAAG
NdespoIVA5'	forward primer for cloning of C-terminal region of *spoIVA* into pETDuet	TCGATCCATATGATCAAAGTAGATG
NdespoIVA3'	reverse primer for cloning of N-terminal region of *spoIVA* into pETDuet	TACTTTGATCATATGGATCGACG

cotEpETDuet5'	forward primer for cloning of region of *cotE* into pETDuet	GAGAGAGCATATGTCTGAATACA GGGAAATTATTACG
cotEpETDuet3'	reverse primer for cloning of *cotE* into pETDuet	GAGAGAGCTCGAGTTATTCTTCAG GATCTCCCAC

Table S2. Plasmids and strains.

Name	Description	Reference
pUT18	BATCH vector for expression of a protein fused with T18 fragment of adenylate cyclase at its N-terminus, Amp^r	[1]
pUT18C	BATCH vector for expression of a protein fused with T18 fragment of adenylate cyclase at its C-terminus, Amp^r	[1]
pKT25	BATCH vector for expression of a protein fused with T25 fragment of adenylate cyclase at its N-terminus, Km^r	[1]
pKNT25	BATCH vector for expression of a protein fused with T25 fragment of adenylate cyclase at its C-terminus, Km^r	[1]
pUT18spoVM	Plasmid expressing *spoVM-T18*, Amp^r	This work
pUT18CspoVM	Plasmid expressing *T18- spoVM,* Amp^r	This work
pKT25spoVM	Plasmid expressing *T25- spoVM,* Km^r	This work
pKNT25spoVM	Plasmid expressing *spoVM-T25*, Km^r	This work
pUT18spoIVA	Plasmid expressing *spoIVA-T18*, Amp^r	This work
pUT18CspoIVA	Plasmid expressing *T18-spoIVA* Amp^r	This work
pKT25spoIVA	Plasmid expressing *T25-spoIVA,* Km^r	This work
pKNT25spoIVA	Plasmid expressing *spoIVA-T25*, Km^r	This work
pUT18yncD	Plasmid expressing *yncD-T18*, Amp^r	This work
pUT18CyncD	Plasmid expressing *T18-yncD,* Amp^r	This work
pKT25yncD	Plasmid expressing *T25-yncD,* Km^r	This work
pKNT25yncD	Plasmid expressing *yncD-T25*, Km^r	This work
pUT18yhaX	Plasmid expressing *yhaX-T18*, Amp^r	This work
pUT18CyhaX	Plasmid expressing *T18-yhaX,* Amp^r	This work
pKT25yhaX	Plasmid expressing *T25-yhaX,* Km^r	This work
pKNT25yhaX	Plasmid expressing *yhaX-T25*, Km^r	This work
pUT18yheD	Plasmid expressing *yheD-T18*, Amp^r	This work
pUT18CyheD	Plasmid expressing *T18 yheD,* Amp^r	This work
pKT25yheD	Plasmid expressing *T25-yheD,* Km^r	This work
pKNT25yheD	Plasmid expressing *yheD-T25*, Km^r	This work
pUT18spoVB	Plasmid expressing *spoVB-T18*, Amp^r	This work
pUT18CspoVB	Plasmid expressing *T18-spoVB,* Amp^r	This work
pKT25spoVB	Plasmid expressing *T25-spoVB,* Km^r	This work
pKNT25spoVB	Plasmid expressing *spoVB-T25*, Km^r	This work
pUT18spoVD	Plasmid expressing *spoVD-T18*, Amp^r	This work
pUT18C spoVD	Plasmid expressing *T18-spoVD,* Amp^r	This work
pKT25 spoVD	Plasmid expressing *T25-spoVD,* Km^r	This work
pKNT25 spoVD	Plasmid expressing *spoVD-T25*, Km^r	This work

pUT18spoVE	Plasmid expressing *spoVE-T18*, Amp^r	This work
pUT18CspoVE	Plasmid expressing *T18-spoVE*, Amp^r	This work
pKT25spoVE	Plasmid expressing *T25-spoVE*, Km^r	This work
pKNT25spoVE	Plasmid expressing *spoVE-T25*, Km^r	This work
pUT18cotE	Plasmid expressing *cotE-T18*, Amp^r	[2]
pUT18CcotE	Plasmid expressing *T18-cotE*, Amp^r	[2]
pKT25cotE	Plasmid expressing *T25-cotE*, Km^r	[2]
pKNT25cotE	Plasmid expressing *cotE-T25*, Km^r	[2]
E. coli BTH101	F^-, cya-99, araD139, galE15, galK16, rpsL1 (Str r), hsdR2, mcrA1, mcrB1	Euromedex
E. coli DH5*α*	F^- Φ80*lac*ZΔM15 Δ(*lac*ZYA-*arg*F) U169 *rec*A1 *end*A1 *hsd*R17 (rK–, mK+) *pho*A *sup*E44 λ– *thi*-1 *gyr*A96 *rel*A1	Lab stock
Bacillus subtilis	PY79	[3]
LMD104	trpC2 DspoVD amyE::PspoVD-spoVD-mCherry; SpR	[4]
BKK15810	trpC2 ΔspoVM::kan	[5]

References

1. Karimova, G.; Pidoux, J.; Ullmann, A.; Ladant, D. A bacterial two-hybrid system based on a reconstituted signal transduction pathway. Microbiology 1998, 95, 5752–5756.

2. Qiao, H.; Krajcikova, D.; Xing, C.; Lu, B.; Hao, J.; Ke, X.; Wang, H.; Barak, I.; Tang, J. Study of the interactions between the key spore coat morphogenetic proteins CotE and SpoVID. J. Struct. Biol. 2013, 181, 128–135, doi:10.1016/j.jsb.2012.11.002.

3. Youngman, P.; Perkins, J.B.; Losick, R. Construction of a cloning site near one end of Tn917 into which foreign DNA may be inserted without affecting transposition in Bacillus subtilis or expression of the transposon-borne erm gene. Plasmid 1984, 12, 1–9, doi:10.1016/0147-619X(84)90061-1.

4. Bukowska-Faniband, E.; Hederstedt, L. Cortex synthesis during Bacillus subtilis sporulation depends on the transpeptidase activity of SpoVD. FEMS Microbiol. Lett. 2013, 346, 65–72, doi:10.1111/1574-6968.12202.

5. Koo, B.; Kritikos, G.; Farelli, J.D.; Todor, H.; Tong, K.; Kimsey, H.; Wapinski, I.; Galardini, M.; Cabal, A.; Peters, J.M.; et al. Construction and analysis of two-genome scale deletion libraries for Bacillus subtilis. Cell Syst. 2018, 4, 291–305, doi:10.1016/j.cels.2016.12.013.
